# Alzheimer's disease models and functional genomics—How many needles are there in the haystack?

**DOI:** 10.3389/fphys.2012.00320

**Published:** 2012-08-08

**Authors:** Jürgen Götz, Miriam Matamales, Naeman N. Götz, Lars M. Ittner, Anne Eckert

**Affiliations:** ^1^Centre for Ageing Dementia Research, Queensland Brain Institute, The University of QueenslandSt Lucia, QLD, Australia; ^2^Alzheimer's and Parkinson's Disease Laboratory, Brain and Mind Research Institute, University of SydneyCamperdown, NSW, Australia; ^3^Neurobiology Laboratory, Psychiatric University Clinics Basel, University of BaselBasel, Switzerland

**Keywords:** Alzheimer's disease, amyloid, frontotemporal dementia, kinase, phosphatase, proteomic, tau, transcriptomic

## Abstract

Alzheimer's disease (AD) and frontotemporal lobar degeneration (FTLD) are complex human brain disorders that affect an increasing number of people worldwide. With the identification first of the proteins that aggregate in AD and FTLD brains and subsequently of pathogenic gene mutations that cause their formation in the familial cases, the foundation was laid for the generation of animal models. These recapitulate essential aspects of the human conditions; expression of mutant forms of the amyloid-β protein-encoding *APP* gene in mice reproduces amyloid-β (Aβ) plaque formation in AD, while that of mutant forms of the tau-encoding *microtubule-associated protein tau (MAPT)* gene reproduces tau-containing neurofibrillary tangle formation, a lesion that is also prevalent in FTLD-Tau. The mouse models have been complemented by those in lower species such as *C. elegans* or *Drosophila*, highlighting the crucial role for Aβ and tau in human neurodegenerative disease. In this review, we will introduce selected AD/FTLD models and discuss how they were instrumental, by identifying deregulated mRNAs, miRNAs and proteins, in dissecting pathogenic mechanisms in neurodegenerative disease. We will discuss some recent examples, which includes miRNA species that are specifically deregulated by Aβ, mitochondrial proteins that are targets of both Aβ and tau, and the nuclear splicing factor SFPQ that accumulates in the cytoplasm in a tau-dependent manner. These examples illustrate how a functional genomics approach followed by a careful validation in experimental models and human tissue leads to a deeper understanding of the pathogenesis of AD and FTLD and ultimately, may help in finding a cure.

## Introduction

Alzheimer's disease (AD) is a progressive neurodegenerative disease. It is characterized by the functional impairment and loss of neurons, that results in a progressive decline in memory and other cognitive functions, leading to dementia (Ballard et al., 2011). Frontotemporal dementia (FTD) is a related disorder that comprises a group of behavioral, language, and movement disorders. On the basis of the nature of the characteristic protein inclusions, frontotemporal lobar degeneration (FTLD) can be subdivided into the more frequent FTLD-tau and FTLD-TDP subtypes, as well as FTLD-FUS and FTLD-UPS that are less common (Mioshi et al., 2010; Goedert et al., 2012). FTLD-Tau includes Pick's disease (PiD), FTD with Parkinsonism linked to chromosome 17 (FTDP-17), argyrophilic grain disease (AgD), corticobasal degeneration (CBD) and progressive supranuclear palsy (PSP). FTLD is the second most common form of dementia presenting before the age of 65 (Liscic et al., 2008).

It has only been in recent years that it became widely accepted that with an ageing population, AD and related disorders will inevitably reach epidemic proportions. Worldwide numbers are in the order of an estimated 35.6 million. By 2050, this figure will have increased to a projected 115 million. The countries or regions with the largest numbers of affected individuals are China and the developing Western Pacific, Western Europe, and the Unites States (Ballard et al., 2011). The latest figures released by the Alzheimer's Association clearly illustrate the dimension of the problem (Alzheimer's Association. 2012 AD facts and figures. *Alzheimer's and Dementia: The Journal of the Alzheimer's Association*. March 2012; 8:131–168.): 5.4 million Americans are living with AD. Another way to put this figure into perspective is that one in eight older Americans has AD. This disease is the sixth-leading cause of death in the United States and the only cause of death among the top 10 in the United States that cannot be prevented, cured or even slowed. While deaths from AD are dramatically rising (by 66% between 2000 and 2008), deaths from other major diseases such as stroke (−20%), breast cancer (−3%) or prostate cancer (−8%) are dropping. In caring for persons with AD and other dementias, more than 15 million Americans provide unpaid care valued at $210 billion. Payments for care are estimated to be $200 billion in the United States in 2012 alone. These numbers are staggering and can be easily extrapolated to other countries.

The importance of AD is also reflected by the research output in the field. A PubMed search with the term “Alzheimer” identifies approximately 6000 papers in 2011 alone; it is therefore impossible to provide a comprehensive overview of the field. Nonetheless, as this review article is part of a series aimed at covering systems biology approaches (e.g., to understand the role of kinases) in diseases such as AD, it is important to put the systems biology approaches in AD and also the role of kinases in the pathogenesis of AD into perspective. Starting with the initial discovery of Alois Alzheimer of the key histopathological features of a disease that would eventually bear his name, we will provide a short overview of the studies that have lead to the identification of the proteins that make up the hallmark lesions in AD. It will be inevitable to discuss another form of dementia, FTLD-Tau, as it shares important clinical and histopathological features with AD (Jucker and Walker, 2011). We will then discuss those genes, for which pathogenic mutations have been identified in familial cases. Incidentally, they encode the proteins that form the hallmark lesions in AD and FTLD or at least have a role in their formation, strengthening the view that understanding their role in disease is crucial for developing a cure (Goate and Hardy, 2012). Clinically, the familial cases are identical to the more frequent sporadic cases, except that they have an earlier onset. Insight into the disease process has initially been obtained by the analysis of human tissue, but with the identification of pathogenic mutations it has been possible to establish transgenic animal models that express the mutant forms. Modeling has been mainly done in mice, but eventually also in lower species such as the fruitfly *Drosophila melanogaster* or the round-worm *Caenorhabditis elegans* (Gotz and Ittner, 2008). The animal models recapitulate the human pathology and have been instrumental in dissecting underlying pathogenic mechanisms. With this insight, the models were successfully employed in developing treatment strategies that guided clinical trials in human AD patients, such as Aβ-targeted vaccinations (Gotz et al., 2012). As we will discuss in detail, many animal and cellular models were further used to identify deregulated genes, miRNAs and proteins, followed by a functional validation in human tissue (Hoerndli et al., 2005). Likewise, expression data in humans have been validated in experimental systems. In the final part of this review article we will discuss where we believe the AD field is heading and especially the role we expect functional genomics approaches will have in these endeavors.

## From Alois Alzheimer's landmark discovery in 1907 to the last thirty years of using biochemical and molecular techniques

**Figure 1:**
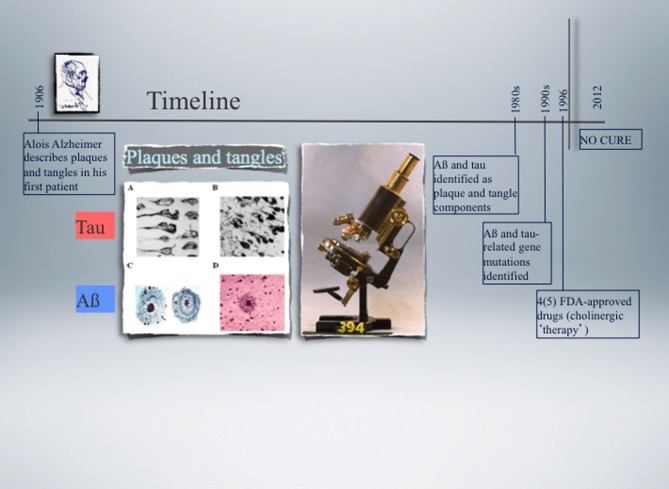
**Timeline of the landmark discovery of Alzheimer plaques and tangles made by Alois Alzheimer in 1906, followed by the identification of Aβ and tau as principal plaque and tangle components, respectively, over 70 years later.** Another decade later, the first pathogenic mutations were identified in the *APP* and *PSEN* genes in Alzheimer's disease (AD) (1989), and nine years later in the *MAPT* gene in a subset of cases with frontotemporal dementia (FTD) (1998). APP encodes the amyloid precursor protein from which Aβ is derived by proteolytic cleavage, and *PSEN* encodes presenilin, one of four component of the secretase complex that generates Aβ. Tau is a microtubule-associated protein encoded by the *MAPT* gene. The microscope used by Alzheimer is shown as well as representative images of plaques and tangles. None of the currently available drugs prevent or delay the neuronal degeneration that characterises AD and FTD as they do not target the underlying biology. Functional genomics approaches in experimental cellular and animal systems are expected not only to contribute to a better understanding of how Aβ and tau cause neuronal demise, but also to assist in developing more effective therapies.

2006 marked the 100th anniversary of a lecture the German psychiatrist and neuropathologist Alois Alzheimer had given in Tübingen, a city in the Southern part of Germany, presenting the clinical and neuropathological characteristics of the disease that his colleague Emil Kraepelin would subsequently name after him (Alzheimer et al., 1995). The year of this anniversary saw many excellent reviews highlighting the initial discoveries and what the scientific community has achieved in the past hundred years (Goedert and Spillantini, 2006; Roberson and Mucke, 2006). It was in November 1901 that Alzheimer admitted Auguste D., a 51-year-old patient, to the Frankfurt hospital because of progressive memory loss, focal symptoms, delusions, and hallucinations. When Auguste D. died in April 1906, her brain was sent to Munich for a histopathological analysis. Alzheimer used a silver staining method developed by Max Bielschowsky a few years earlier that is still in use today. This method was crucial for him to identify the two key defining neuropathological characteristics of AD, the neuritic plaques and the neurofibrillary tangles (Figure 1). While plaques had been reported before in a patient with epilepsy, Alzheimer was the first to describe the tangle pathology. A few years later, he also discovered another type of (spherical) lesion now known as Pick body. The corresponding disease was named PiD, after Arnold Pick, who first described it in 1892 (Goedert and Spillantini, 2006). PiD belongs to the spectrum of FTLD. While the presence of abnormal deposits helped greatly with disease classification (Blessed et al., 1968), it was only during the past thirty years that their molecular composition and role in the pathological process was elucidated.

The plaques are extracellular deposits, whereas the tangles form intraneuronally. The two lesions have the filamentous nature of their respective protein components in common, and both types of filaments can be visualised by electron microscopy. After they were first described in the early 1960s, it took another twenty years until their major constituents were identified, amyloid-β (Aβ) as the principal plaque component, and tau as the principal tangle component (Figure 1). Aβ has a size of mainly 40–42 amino acids and is derived by proteolytic cleavage from the larger amyloid precursor protein, APP (Glenner and Wong, 1984; Masters et al., 1985). β-Secretase generates the amino-terminus of Aβ and γ-secretase dictates its length, with Aβ40 being the more common and Aβ42 the more fibrillogenic and neurotoxic species. Aβ forms toxic oligomeric aggregates and eventually deposits as plaques. Additional products of APP processing are an amino-terminal fragment that is released by shedding, and AICD, the Aβ intracellular cytoplasmic domain. β-Secretase activity has been attributed to a single protein, BACE (Vassar et al., 1999), whereas γ-secretase activity depends on four components, presenilin, nicastrin, APH-1 and PEN-2 (Edbauer et al., 2003). α-Secretase is involved in the non-amyloidogenic pathway by cleaving APP within the Aβ domain, thus precluding Aβ formation (Lammich et al., 1999). More recent studies identified additional species of Aβ, such as pyroglutamate-modified forms of Aβ or species ranging from 39 to 43 amino acids, that all to some degree or another are believed to have a role in disease initiation (Saito et al., 2011; Cynis et al., 2008). Historically, it was the mapping of the APP gene to chromosome 21, together with the observation of plaques and tangles in most elderly individuals with Down's syndrome (trisomy of chromosome 21) followed by the linking of some cases of familial AD to chromosome 21 (Goate et al., 1989; St George-Hyslop et al., 1990), that lead to the identification of the first mutations in the *APP* gene (Chartier-Harlin et al., 1991; Goate et al., 1991; Murrell et al., 1991) (Figure 1). Since then, more than 20 pathogenic mutations have been identified, they however account for only a minority of familial AD cases. Most mutations are in the *presenilin 1 (PSEN1)* and *PSEN2* genes, and until today, over 130 mutations have been identified (http://www.molgen.ua.ac.be/admutations/). The familial cases altogether account for less than 1%. Of the many susceptibility or risk genes identified in AD, *apolipoprotein E (ApoE)* is the most important one (Kim et al., 2009). The human *ApoE* gene contains several single-nucleotide polymorphisms (SNPs) that are distributed across the gene (Nickerson et al., 2000). The most common three SNPs lead to changes in the coding sequence and result in the three common isoforms of apoE: apoE2 (Cys112/Cys158), apoE3 (Cys112/Arg158), and apoE4 (Arg112/Arg158). Although they differ by only one or two amino acids at residue 112 or 158, these differences alter the apoE structure and function profoundly (Mahley et al., 2006). The increased risk for AD is 2- to 3-fold in people with one *ApoE4* allele, and about 12-fold in individuals with two *ApoE4* alleles (Roses, 1996). In fact, as 64% of sporadic AD cases are *ApoE4* carriers, this allele comprises a major risk factor in AD.

As for Aβ, it took several years until it was recognized that the filaments that make up the neurofibrillary tangles are composed of hyperphosphorylated forms of the protein tau (Brion et al., 1985; Delacourte and Defossez, 1986; Grundke-Iqbal et al., 1986; Ihara et al., 1986; Kosik et al., 1986; Goedert et al., 1988; Wischik et al., 1988) (Figure 1). This protein exists in the murine brain as three major splicing isoforms that all have four microtubule-binding repeats (4R) in common and contain either no, one or two amino-terminal inserts, termed E1 and E2 (creating the isoforms 0N/4R, 1N/4R, and 2N/4R), whereas, in humans, there is, in addition, a set of three 3R isoforms, resulting in a total of six major isoforms (Gotz et al., 2010). Tau is enriched in neurons, but it is also expressed in other cell-types such as oligodendrocytes where it is required for the outgrowth of cellular processes and the kinase Fyn-dependent myelination of neurons (Klein et al., 2002). In the course of neuronal maturation, tau segregates into the axon, and the family member MAP2 into the dendrites (Matus, 1990).

Tau belongs to the family of microtubule-associated proteins (MAPs) (Dehmelt and Halpain, 2005), with different models being put forward to explain how it might bind to microtubules (Al-Bassam et al., 2002; Magnani et al., 2007). Although tau is widely considered as an axonal protein, it has been suggested that a physiological role must exist for tau outside the axonal compartment (Loomis et al., 1990). The view is slowly emerging that tau is in fact an important scaffolding protein with a range of functions in the physiological context (Bi et al., 2011; Ittner and Gotz, 2011; Gotz et al., 2012). For example, we showed that under normal conditions tau is found albeit at low levels in dendrites (Ittner et al., 2010). We further found that tau targets the kinase Fyn to the dendritic compartment, with this kinase phosphorylating the NMDA receptor and thereby mediating its interaction with the post-synaptic density protein 95 (PSD-95) (Ittner and Gotz, 2011). Tau has also been detected on ribosomes of both neuronal and glial cells (Papasozomenos and Su, 1991). We found further that tau affects mitochondrial functions mainly via complex I of the respiratory chain (David et al., 2006; Eckert et al., 2008; Rhein et al., 2009; Eckert et al., 2010). After tau was first identified in 1975 (Weingarten et al., 1975), tau research mainly investigated physiological functions. However, the focus shifted radically when tau was identified as the filamentous core of the neurofibrillary tangles. Insoluble tau aggregates are present in many diseases other than AD, collectively termed tauopathies, where they occur in the absence of overt Aβ deposition (Gotz and Ittner, 2008). Tau has an unusually high content of 84 putative phosphorylation sites, and under pathological conditions, the protein becomes “hyperphosphorylated”, i.e., some sites become phosphorylated to a higher degree in the diseased than in the healthy brain; others are *de novo* phosphorylated (Chen et al., 2004a). These phosphorylation events are required for tau to detach from the microtubules, tau is relocalized from the axon to the somatodendritic compartment, and as soluble tau levels increase, it interacts with cellular proteins and prevents them from executing their normal functions (Ittner et al., 2009; Stoothoff et al., 2009; Ittner et al., 2010). While in AD, no mutations have been identified in the *MAPT* gene encoding tau, until today, over 40 intronic and exonic mutations have been identified in FTD with Parkinsonism linked to chromosome 17 (FTDP-17), a form of FTLD-Tau (Hutton et al., 1998; Poorkaj et al., 1998; Spillantini et al., 1998) (Figure 1). This established that dysfunction of tau by itself can cause neurodegeneration and lead to dementia. A second major subset of FTLD is characterized by lesions that are tau-negative and ubiquitin-positive. In this subset, the transcription and splicing factor TDP-43 (TAR DNA-binding protein 43) was identified as the aggregating protein and hence, this form of disease was termed FTLD-TDP (Neumann et al., 2006). Similar to tau, TDP-43 in the aggregates is hyperphosphorylated and fragmented, a process believed to be linked to toxicity (Neumann et al., 2006; Zhang et al., 2007; Dormann et al., 2009; Igaz et al., 2009). Finally, in a third subset of FTLD, FTLD-FUS, a second nuclear protein, FUS (Fused-in-Sarcoma), was identified as the aggregating protein (Urwin et al., 2010). While both proteins form aggregates also in subsets of amyotrophic lateral sclerosis (ALS), the mechanisms causing disease are believed to differ (Dormann and Haass, 2011). Interestingly, while in FTLD-tau tau is relocalized from the axon to the somatodendritic domain, in FTLD-TDP and -FUS, there is a nucleo-cytoplasmic relocalization of TDP-43 and FUS, respectively. What causes these proteins to form aggregates in the first place is not understood nor why, e.g., in one disease it is FUS that is aggregating while in another disease the aggregating protein is tau.

Of the many post-translational modifications tau undergoes, phosphorylation seems to be the most important one, pointing at the deregulation of kinases and phosphatases as being crucial in the pathogenesis of AD (Chen et al., 2004a). Tau not only contains an unusually high content of putative phosphorylation sites (45 serines, 35 threonines and 4 tyrosines for the longest 441 amino acid human tau isoform, htau40)—it is also highly phosphorylated under physiological conditions, having on average 2–3 moles of phosphate per mole of tau, while under pathological conditions this ratio is increased to 7–8 moles (Kopke et al., 1993). This pathological change has been termed “hyperphosphorylation” (Pettegrew et al., 1987): some sites are phosphorylated to a higher degree in the diseased compared with the healthy brain, while others are *de novo* phosphorylated. Hyperphosphorylation is critical for tau to detach from microtubules and believed to be a prerequisite for aggregation (Avila, 2006). Potential therapeutic strategies are directed at a wide variety of kinases, including glycogen synthase kinase 3β (GSK3β), cyclin-dependent kinase 5 (Cdk5), JNK, and microtubule-associated regulatory kinase (MARK) (Dolan and Johnson, 2010). Kinase activities are antagonized by phosphatases and for tau they include protein phosphatase 2A (PP2A) and protein phosphatase 2B (PP2B) that is also known as calcineurin (Kins et al., 2003). PP2A binds to tau directly, and this binding is mediated by tau's microtubule-binding domain (Sontag et al., 1996; Xu et al., 2008). Below we will discuss studies addressing the complex role of these enzymes in AD and how they interact.

## Genetic and biochemical discoveries pave the way for animal model development

While significant insight into disease mechanisms has been obtained in invertebrate species such as the roundworm *C. elegans* or the fruitfly *D. melanogaster*, the mouse has been and still is the major species used in AD and FTLD research (Gotz and Gotz, 2009). With the identification of the first pathogenic mutations in the APP gene in familial cases of AD, a plethora of APP mutant mouse strains has been generated, with prominent examples being strains such as PDAPP, J20, APP23, or Tg2576, all of which are characterized by a robust Aβ plaque pathology (Games et al., 1995; Hsiao et al., 1996; Sturchler-Pierrat et al., 1997; Mucke et al., 2000). Representative models with neurofibrillary tangle formation include strains such as the JNPL3 or our pR5 mice, both of which express P301L mutant tau that is found in familial cases of FTDP-17 (Lewis et al., 2000; Gotz et al., 2001a). In the mouse models, Aβ in the plaques is fibrillar, as is tau in the tangles; and in both instances, the histopathology is associated with behavioral impairment (Gotz and Ittner, 2008). In plaque-forming APP23 mice, the age-dependent cognitive decline has been found to precede amyloid deposition (Van Dam et al., 2003). In tangle-forming pR5 mice, owing to a pronounced pathology in the amygdala and hippocampus (Deters et al., 2008), the behavioral impairment was found to correlate with tau aggregation rather than tangle formation which occurred only in a relatively small subset of neurons (Pennanen et al., 2004, 2006).

There is a crosstalk between glucose metabolism (that is deregulated in another epidemic, diabetes mellitus) and the AD pathology as illustrated in mice: when APP23 mice were crossed with diabetic ob/ob mice to establish double-mutant mice, the onset of diabetes was found to exacerbate the cognitive dysfunction already at 8 weeks of age and without an increase in brain amyloid-beta burden (Takeda et al., 2010). In pR5 mice, in comparison, inducing diabetes by injecting streptozotocin increased tau's hyperphosphorylation and insolubility, and accelerated tangle formation arguing that diabetes can accelerate the onset and increase severity of disease in individuals with a predisposition to developing tau pathology (Ke et al., 2009).

While the pR5 mice display memory impairment as a major clinical feature of AD, another feature, Parkinsonism, that characterizes a significant subset of FTLD cases, has been modeled in K369I mutant tau transgenic K3 mice. We established this strain based on the identification of the K369I mutation of tau in a single patient with PiD (Neumann et al., 2001), and reproduced the distinct characteristics of Pick's pathology in mice (Ittner et al., 2008). Memory functions were impaired as shown in the novel object recognition test. Owing to a unique expression pattern of the transgene that extends to the substantia nigra, the K3 mice also model early-onset Parkinsonism, i.e., resting tremor, bradykinesia, postural instability, and gait anomalies. They show an increased cataleptic response to haloperidol and an early, but not late response to L-Dopa, indicating that the dopaminergic system is impaired. We found a selectively impaired axonal transport of distinct cargos including mitochondria and TH (tyrosine hydroxylase)-containing vesicles. At the molecular level, this is caused by trapping of the adapter protein Jip1, a component of the kinesin motor machinery, by elevated levels of phosphorylated tau, preventing Jip1 from executing its physiological function in the axon (Ittner et al., 2008). A pathological interaction between tau and JIP1 was further revealed in AD and not, control brain, highlighting the validity of transgenic animal models in dissecting pathomechanisms in AD (Ittner et al., 2009). We have been using the K3 mice for an ENU mutagenesis screen as outlined below.

Several aspects of the human AD and FTLD pathology have been successfully modeled in the nematode *C. elegans* (Morcos and Hutter, 2009). This roundworm has a number of features that make it a powerful research tool, complementing, but not replacing the mouse as a model organism: (1) it is easy to culture as it feeds of bacteria grown on agar plates; (2) it reproduces and develops rapidly: within 3 days it develops from an egg to an adult worm, with about 300 progenies originating from one self-fertilized hermaphrodite; (3) its small size allows assays in microtitre format, studying hundreds of animals in a single well; (4) the worm is transparent, which is ideal for the use of fluorescent markers *in vivo*; (5) although it is a complex multicellular animal, an adult hermaphrodite has only 959 somatic cells that form all the organs, including 302 neurons that form the nervous system; (6) it has a short live span of 2–3 weeks, allowing aging studies within a reasonable time frame; and (7) genetic modifications, such as transgenic expression or RNAi-mediated gene knockdown are relatively easy compared to other *in vivo* systems. Furthermore, most human disease genes and pathways are present in *C. elegans* (Kaletta and Hengartner, 2006). This includes the *APP* homologue *APL-1*, a mutation of which results in early larval lethality (Hornsten et al., 2007), which can be rescued by a carboxy-terminal truncation suggesting that the extracellular region of the protein is essential for viability (Wiese et al., 2010). These similarities prompted investigators to structurally characterize the E2 domain of APL-1 and draw conclusions for the still elusive physiological function of its mammalian homolog APP in binding to the heparan sulfate proteoglycans and hence, adhesion (Hoopes et al., 2010). *C. elegans* has two presenilin homologs, *SEL-12* and *HOP-1* (Levitan and Greenwald, 1995; Li and Greenwald, 1997), and a single MAP called ptl-1 (protein with tau-like repeats) that is the homolog of both tau and MAP2 (Goedert et al., 1996; McDermott et al., 1996).

*C. elegans* has been successfully used as a model organism to study pathomechanisms in AD and FTLD. Expression of human tau carrying FTLD mutations, but not wild-type tau, results in neurodegeneration with an accumulation of hyperphosphorylated tau and associated uncoordinated locomotion (Unc) (Kraemer et al., 2003; Miyasaka et al., 2005; Brandt et al., 2009). *C. elegans* is not capable of producing endogenous Aβ. When the first published transgenic model targeted Aβ to the body wall of muscle cells, this caused progressive paralysis (Link, 1995). Aβ deposition in another model was associated with oxidative stress (Drake et al., 2003). A further aspect linked to oxidative stress that is evident from studies in *C. elegans* is the similarity between diabetes mellitus and AD, in particular with regards to the formation of advanced glycation end products (Morcos and Hutter, 2009). Taking epidemiological studies a step further that had suggested that caffeine consumption may reduce the AD risk, it was found by screening in worms that it is not caffeine that is protective but rather the activation of the highly conserved skn-1/Nrf2 detoxification pathway (Dostal et al., 2010). An interesting recent study looked at the role of protein homeostasis in longevity (Alavez et al., 2011). *C. elegans* is a preferred system to study ageing and genetic determinants in longevity which again informs on susceptibility to neurodegeneration (Freude et al., 2009; Reis-Rodrigues et al., 2012). It was found that small molecules such as thioflavin T (ThT), a dye traditionally used in histopathology to stain amyloid in tissues, not only slowed protein aggregation in vitro and in cell culture, but also profoundly extended the lifespan and slowed ageing in *C. elegans* via regulators of protein homeostasis (Alavez et al., 2011). Together this demonstrates the potential of *C. elegans* as a model organism in AD and FTLD research.

The findings obtained in the worm are complemented by studies in Drosophila, a powerful system to screen for modifiers that either enhance or suppress an AD-associated pathology (Shulman and Feany, 2003; van de Hoef et al., 2009). Drosophila has been instrumental in determining the role of phosphorylation in tau aggregation (Steinhilb et al., 2007) and in providing direct evidence for DNA damage and checkpoint activation in tauopathies (Khurana et al., 2012). Interestingly, the pathological changes in the mouse are mirrored by those found in the experimentally more accessible worm. Together this raises the possibility of a generally protective function for the DNA damage checkpoint in diseases of aging.

## The advent of functional genomics—support for prevailing hypotheses and identification of new targets

With the advent of multiplex techniques in functional genomics, both transcriptomic and proteomic approaches have been increasingly applied in the field (Hoerndli et al., 2005; David et al., 2005a). As the currently prescribed drugs are symptomatic (Figure 1) and altogether over two dozen clinical trials have failed, one of the hopes is that the screening efforts will not only contribute to a basic pathogenic understanding but may also help in identifying new drug targets, thereby complementing the newer treatment strategies that aim to reduce the levels of either Aβ or hyperphosphorylated tau (Ballard et al., 2011). Functional genomics approaches have been particularly helpful in determining the downstream toxic consequences of increased Aβ and tau levels and target these for therapeutic intervention. Not surprisingly, category analysis identified some usual suspects and pathways, however, as we will outline, they also identified novel potential drug targets. One of the major challenges resides in determining what the relative contribution is of the different molecules and mechanisms, as some of the deregulated genes and proteins have roles in general mechanisms such as axonal transport, the MAPK signaling pathway, mitochondrial function, cell cycle regulation, and so forth (Gotz and Gotz, 2009). Only slowly it is becoming clear what the relative role is of Aβ and tau in causing neuronal toxicity in the first place, and therefore it will take a while until the role of their down-stream mediators will be unambiguously determined (Gotz et al., 2008). Obviously, with therapy in mind, one might also regulate Aβ and tau directly, either at the transcriptional, translational or post-translational level. Here, miRNAs are newly emerging therapeutic tools (Zovoilis et al., 2011).

In AD not all brain areas are affected equally by degeneration. In fact, the progression of lesions such as of the neurofibrillary tangles has led to the definition of the so-called Braak stages, as tangle formation is initiated in the entorhinal cortex and spreads from there in a stereotypic fashion to the hippocampus and eventually to anatomically linked higher cortical brain regions (Braak and Braak, 1991). This has led to coining of the term “selective vulnerability” raising the possibility that because of a specific genetic “make-up” some brain areas are more prone to degeneration while others are relatively resistant (Gotz et al., 2009). By assessing the brain of experimental species such as mice, an essential question therefore is to which extent there is brain region-specific gene expression in the first place. When the hippocampal subregions CA1, CA3, and DG of wild-type mice were analysed by gene arrays and qRT-PCR, the maximal difference observed for any gene was 7.6-fold and none of the genes seemed to be transcribed exclusively in any of these subregions (Zhao et al., 2001). In a related study using gene arrays and Northern blotting of cortex, cerebellum and midbrain, less than 1% of the genes showed a clear enrichment and for the cerebellum, for example, only 14 genes showed a restriction or high expression, while 14 other genes were completely absent (Sandberg et al., 2000). Interestingly, different from the cerebellum, the structures of the medial temporal lobe (i.e., hippocampus, amygdala and entorhinal cortex) showed very similar expression profiles and only eight genes were unique to one of the three regions. This would suggest that forebrain structures, despite some functional differences, are highly similar at the molecular level (Sandberg et al., 2000). It has to be kept in mind that with the more sensitive methods that are available today (such as high density arrays and in particular RNA sequencing) these numbers may change drastically (Ramskold et al., 2012). In two complementary studies, the amygdala has been shown to be a hotspot for Aβ-mediated tau tangle formation in mice suggesting a role for differential gene expression in vulnerability to Aβ (Lewis et al., 2001; Gotz et al., 2001b). Furthermore, the behavioral impairment was correlated with the presence of tau aggregates in distinct subnuclei of the amygdala (Pennanen et al., 2004). In support, a microarray analysis found that although in wild-type mice on average only 0.3% of the 34,000 genes interrogated were highly enriched in each of the five regions analysed (that included the amygdala), *in situ* hybridization performed on a subset of the amygdala-enriched genes mostly confirmed the overall region-specificity predicted by the microarray data, revealing boundaries of expression within the amygdala that corresponded to cytoarchitectonically defined subnuclei (Zirlinger et al., 2001). In AD, however, selective vulnerability in AD may be of a multigenic nature. Nonetheless, distinct genes with protective function may have the potential of being exploited for therapeutic intervention (Greeve et al., 2000).

Several hypotheses have been put forward to explain neurodegeneration in AD. While the amyloid cascade hypothesis has had a major impact in the field [by placing Aβ in a pathocascade upstream of tau (Hardy, 2006)], work from several groups revealed an essential role for tau in mediating Aβ toxicity (Ittner et al., 2010; Rapoport et al., 2002; Roberson et al., 2007). More specifically, removing tau from the dendritic compartment rescued Aβ plaque-forming mice from increased lethality, impaired memory functions and increased susceptibility to excitotoxic seizures (Roberson et al., 2007; Ittner et al., 2010; Ittner and Gotz, 2011). Excitotoxicity has been implicated in AD, leading to excessive nitric oxide (NO) levels, which causes down-stream protein misfolding and aggregation, as well as oxidation damage of mitochondria. Support for the oxidation damage hypothesis that overlaps with the impaired axonal transport hypothesis is provided by functional genomics approaches in both cellular and animal models.

Not surprisingly, oxidative stress and mitochondria are gaining increasing attention in diseases affecting the nervous system (Marazziti et al., 2012). Mitochondria are dynamic organelles that move from the cell body to regions of the cell to deliver ATP and other metabolites where they are most required, and then return. This is seen most strikingly in highly elongated cells such as neurons: mitochondria are enriched at presynaptic terminals at the ends of axons and at post-synaptic terminals at the ends of dendrites, where the bioenergetic demand is particularly high (Schon and Przedborski, 2011). A recent study provides transcriptomic and proteomic evidence that the neuronal nuclear genes influencing mitochondrial energy metabolism are underexpressed in AD, particularly in brain regions like the posterior cingulate cortex, which are found to be preferentially affected in PET studies of AD patients and cognitively normal persons at genetic risk for this disorder (Liang et al., 2008). A MALDI TOF/TOF mass-spectrometric analysis of P301L tau transgenic pR5 mice revealed a deregulation of mainly metabolic-related proteins including mitochondrial respiratory chain complex components (including the complex V component ATP synthase D chain), antioxidant enzymes and synaptic proteins (David et al., 2005b). Deregulated kinases were not observed possibly reflecting generally low protein levels of these enzymes. However, indirect changes were found such as decreases of GRB2, an adapter protein involved in the activation of the mitogen-activated protein kinase signaling pathway. A subsequent functional analysis demonstrated a mitochondrial dysfunction in pR5 mice together with reduced NADH-ubiquinone oxidoreductase activity and, with age, impaired mitochondrial respiration and ATP synthesis. Mitochondrial dysfunction was associated with higher levels of reactive oxygen species in aged transgenic mice and an up-regulation of antioxidant enzymes. When complex V levels were analyzed in human FTDP-17 patient brains carrying the P301L tau mutation a significant decrease was found in all P301L brain samples compared to controls underscoring the validity of the proteomics findings for the human disease. Importantly, pR5 mitochondria displayed an increased vulnerability towards Aβ, suggesting a synergistic action of tau and Aβ pathology on the mitochondria (David et al., 2005b). A serial analysis of gene expression (SAGE) of the same mouse strain looking specifically at the amygdala also revealed deregulated mitochondrial genes (Ke et al., 2012). Furthermore, the β catalytic subunit of protein phosphatase 3 (encoded by *Ppp3cb*) was 2-fold down-regulated in the pR5 mice, while CDC-like kinase 1 was more than 4-fold up-regulated (Ke et al., 2012). When vesicular fractions were isolated from Aβ plaque-forming Tg2576 mice, and proteins separated by 2-D DIGE and identified by mass spectrometry followed by a functional validation, this identified numerous changes in the protein subunit composition of the respiratory chain complexes I and III, impaired state 3 respiration and uncoupled respiration (Gillardon et al., 2007), similar to what has been found in P301L tau transgenic pR5 mice (David et al., 2005b) and Aβ plaque-forming Thy-1.APP transgenic mouse strain (Hauptmann et al., 2008). As this impairment occurred before NFT formation and Aβ plaque deposition it suggests that mitochondria are early targets of Aβ and tau aggregates (Eckert et al., 2011). To address the cross-talk of Aβ and tau on mitochondrial functions, pR5 mice were bred with Aβ plaque-forming APP^sw^PS2^N141I^ double-transgenic APP152 mice to generate triple transgenic (^triple^AD) mice that combine both pathologies in one model (Grueninger et al., 2010). Quantitative mass-tag labeling (iTRAQ) followed by mass spectrometry revealed a massive deregulation of 24 proteins of which one third were mitochondrial proteins mainly related to complexes I and IV of the oxidative phosphorylation system (OXPHOS). Notably, deregulation of complex I was tau-dependent, while deregulation of complex IV was Aβ-dependent, both at the protein and activity levels (Rhein et al., 2009). In addition, synergistic effects of Aβ and tau were evident in the ^triple^AD mice establishing a molecular link between Aβ and tau protein in AD pathology *in vivo* (Rhein et al., 2009). In the mice, phosphorylation of tau at the epitope Ser422 was enhanced (Grueninger et al., 2010) and incidentally, this is an epitope that has been tightly linked to tau tangle formation (Gotz et al., 2001b; Ferrari et al., 2003). To determine the kinase responsible for Ser422 phosphorylation of tau, screening of a library of 65,000 kinase inhibitors was combined with *in vitro* inhibitor target profiling of the screening hits using the Ambit kinase platform: this identified the kinase MKK4 (Grueninger et al., 2011). MKK4 is a regulatory kinase and very probably controls Ser422 phosphorylation of tau via activation of one of its immediate downstream target kinases (Grueninger et al., 2011). To date only two kinases have been described as MKK4 substrates, namely P38 and JNK, and both these kinase families are known to phosphorylate tau at Ser422 *in vitro* (Goedert et al., 1997).

Oxidation damage, inflammation and stress response are tightly linked. When the CA1 region from AD cases was compared to control subjects, an Affymetrix analysis mainly revealed an up-regulation of oxidative stress-related, apoptosis-related and pro-inflammatory signaling genes (Colangelo et al., 2002); a comparative analysis of three APP mouse models by gene arrays identified mainly deregulated genes with roles in the immune response, carbohydrate metabolism, and proteolysis; while in P301L tau transgenic JNPL3 mice, inflammation mediators and apoptosis inhibitors were found to be down-regulated (Ho et al., 2001). In the P301L tau transgenic pR5 mice, glyoxalase I was the only up-regulated gene when a stringent analysis was applied (Chen et al., 2004b). This enzyme plays a critical role in the detoxification of dicarbonyl compounds and thereby reduces the formation of advanced glycation end products. Levels of both glyoxalase I mRNA and protein were significantly elevated in pR5 brains (Chen et al., 2004b). Co-staining with a phospho-tau antibody suggested glyoxalase I upregulation as an early defense mechanism to combat elevated levels of aggregated tau. When different annotation databases were used to compare these transcriptomic data with the SAGE analysis of RNA extracted from pooled amygdalae of P301L tau transgenic pR5 mice (Ke et al., 2012) it was found by using GenMAPP pathways as annotation database, that the two datasets shared deregulated genes involved in inflammation and proteasome degradation (Hoerndli et al., 2004). To understand which processes are disrupted by Aß42 in the presence of tau aggregates, comparative proteomics was also applied to Aß42-treated P301L tau expressing neuroblastoma cells and the amygdala of P301L tau transgenic pR5 mice stereotaxically injected with Aß42: A significant fraction of proteins altered in both systems belonged to the same functional categories, i.e., proteins involved in the stress-response associated with protein folding (David et al., 2006). Among the deregulated proteins was VCP (valosin containing protein), an essential component of the ER-associated degradation (ERAD) process. Down-regulated were also members of the peroxiredoxin family. For comparison, over-expression of Aβ and amyloid binding alcohol dehydrogenase (ABAD), an intracellular binding site for Aβ, in transgenic mice identified deregulated peroxiredoxin 2 levels a protein subsequently shown to be increased in the AD brain. The authors argued that the expression level of peroxiredoxin 2 is an indicator for the interaction of ABAD and Aβ as its expression levels return to normal when this interaction is perturbed, suggesting Aβ-ABAD interaction as a suitable drug target (Yao et al., 2007). More specifically, the ABAD inhibitor AG18051 has been found to protect SH-SY5Y neuroblastoma cells from Aβ42 toxicity (Lim et al., 2011).

Given the critical role of mitochondria in maintaining cell viability, it stands to reason that defects in mitochondrial trafficking could underlie neurodegenerative processes; however direct evidence that mitochondrial trafficking is altered in patients with neurodegenerative disease is actually quite limited (Schon and Przedborski, 2011). In animal models however, defects in transport have been functionally addressed and there is increasing evidence also from functional genomics studies that hint at axonal transport and mitochondrial dysfunction as central pathomechanisms. In a microarray analysis of tangle-bearing neurons several classes of mRNAs known to encode proteins implicated in AD neuropathology were deregulated, including cytoskeletal and synaptic proteins, as well as glutamate and dopamine receptors (Ginsberg et al., 2000). A few studies focused on early changes associated with mild cognitive impairment (MCI). In one microarray analysis of a small brain sample size, in the superior temporal gyrus down-regulation was found for genes encoding synaptic vesicle proteins such as synapsin IIa, and genes involved in cytoskeletal mobility (Pasinetti, 2001). A follow-up study using proteomics confirmed many of the changes in proteins with synaptic activities (Pasinetti and Ho, 2001). Wild-type mice that had undergone a standard Morris water maze paradigm were used to identify memory-related genes. Of more than 1000 relevant genes, 140 were hippocampal memory-related including genes encoding glutamate receptors, ion channels and also trafficking proteins (Cavallaro et al., 2002). Interestingly, in the adult Down syndrome brain, gene expression profiling identified deregulated genes with roles in processes concomitant with cytoskeletal regulation and vesicle trafficking categories, and an increased immune and oxidative stress response, which are likely linked to the development of AD pathology in individuals with Down syndrome (Lockstone et al., 2007). When a pilot proteomic study was performed in brain samples of patients with AD and normal control subjects to identify APP interactors, 21 proteins were identified which could be grouped into five functional classes: molecular chaperones, cytoskeletal and structural proteins, adaptors, enzymes, and proteins involved in trafficking. Subsequent validation was compatible with an involvement of APP in axonal transport and vesicular trafficking (Cottrell et al., 2005). We established the K369I mutant tau transgenic K3 mice that models memory impairment and Parkinsonism that characterize a significant subset of cases of FTLD (Ittner et al., 2008, 2009). Moreover, the motor phenotype was instrumental in determining tau-mediated impaired axonal transport of distinct cargoes including tyrosine hydroxylase-containing vesicles and mitochondria as underlying pathomechanism. As in the K3 mice the motor phenotype is evident at a very early age, clasping, tremor and postural stability can be used as an easy readout for the efficacy of any intervention be it pharmacological or genetic (van Eersel et al., 2010), or for a modifier screening using ENU mutagenesis (Liu et al., 2011).

A rarely used method to identify differentially expressed genes is the above-mentioned SAGE (Velculescu et al., 1995). The beauty of this method compared to gene chip approaches lies in the fact that it is firstly unbiased and secondly that rather than obtaining an endlessly long list of deregulated genes one ends up with a rather limited number. SAGE works by transcribing RNA into cDNA, followed by digestion with a type IIS restriction enzyme that yield 10–14 base-pair long sequence tags that are annealed followed by sequencing. By sequencing 92,000 tags we identified 29 deregulated genes in the amygdala of pR5 mice compared to controls which included *Sfpq* that encodes a nuclear factor implicated in the splicing and regulation of gene expression (Ke et al., 2012). To assess the relevance for human disease we analyzed brains from the two tauopathies, AD and PiD, as well as control cases. Strikingly, in AD and PiD, affected brain areas showed a virtually complete nuclear depletion of SFPQ in both neurons and astrocytes, along with cytoplasmic accumulation. Accordingly, neurons harboring either AD tangles or Pick bodies were also depleted of SFPQ. Immunoblot analysis of human entorhinal cortex samples revealed reduced SFPQ levels with advanced Braak stages suggesting that the SFPQ pathology may progress together with the tau pathology in AD. To determine a causal role for tau, we stably expressed both wild-type and P301L human tau in human SH-SY5Y neuro-blastoma cells, an established cell culture model of tau pathology (Pennanen and Gotz, 2005). The cells were differentiated by two independent methods, mitomycin C-mediated cell cycle arrest or neuronal differentiation with retinoic acid. Confocal microscopy revealed that SFPQ was confined to nuclei in non-transfected wild-type cells, whereas in wild-type and more pronounced in P301L tau over-expressing cells, irrespective of the differentiation method, it formed aggregates in the cytoplasm, suggesting that pathogenic tau drives SFPQ pathology in post-mitotic cells (Ke et al., 2012). These findings add SFPQ to a growing list of transcription factors with an altered nucleo-cytoplasmic distribution under neurodegenerative conditions highlighting RNA mismanagement as a general pathomechanism (Patel and Chu, 2011).

While a lot of efforts have concentrated on deregulated proteins and protein-encoding genes, more recently significant attention has started focusing on microRNAs (miRNAs). These are cellular gene silencing tools that add another level of complexity to gene regulation. Evolutionary conserved, these 19–24 nucleotide-long non-coding RNAs negatively regulate the expression of specific mRNA targets through base pairing between their “seed region” and sequences commonly located in the 3′ untranslated region of their targets (Fabian et al., 2010; Siomi and Siomi, 2010). In the mouse brain, there are in the order of 300 miRNAs expressed, and each can target up to several hundred to a thousand transcripts (Landgraf et al., 2007). Unfortunately, however, so far only a few targets have been confirmed *in vivo* (Lau and de Strooper, 2010). The recent years have nonetheless seen an explosion in studies linking miRNAs to pathological processes, and evidence is mounting that they have a role in neurodegenerative diseases ranging from AD (Schonrock et al., 2011) to Parkinson's disease (Kim et al., 2007) and ALS (Williams et al., 2009). After discovering the first human miRNAs (Pasquinelli et al., 2000), it took several years until the first report on differential miRNA profiles was published for human AD tissue (Lukiw, 2007). Since then many groups performed genome-wide profiling highlighting specific changes in the miRNA regulatory system in human AD brain (Hebert et al., 2008; Wang et al., 2008; Nunez-Iglesias et al., 2010; Shioya et al., 2010). These may either involve neuronal or glial cells or both, as both cell types are affected in human “neurodegenerative” conditions (Kurosinski and Gotz, 2002). Accumulating data indicate that miRNAs act at several levels: in the case of APP, they regulate *APP* mRNA levels and have a role in alternative splicing. They further regulate APP processing indirectly via the β-secretase BACE1 that has been identified as a miRNA target, with the miRNA cluster containing miR-29a, -29b1, and -9 playing a crucial role (Hebert et al., 2008; Zong et al., 2011). Conversely, Aβ itself has down-stream effects on miRNA expression: when primary hippocampal neurons were incubated with fibrillar preparations of Aβ42, we found that this invoked a strong and rapid change in miRNA profiles with a substantial proportion of miRNAs being down-regulated. miR-9 is the most abundant human brain miRNA (Mattick and Makunin, 2005) and a recurring candidate from several AD profiling studies. We validated this and eight additional miRNAs (miR-181c, -148b, -30c, -20b, -361, -21, -409-3p, and Let-7i) as being down-regulated by Aβ42 (Schonrock et al., 2010). Interestingly, the down-regulation of miRNAs in Aβ-treated hippocampal neurons was paralleled by those in the hippocampus of Aβ-plaque forming APP23 mice at the onset of plaque formation. Another study suggests a possible transient effect of Aβ plaque pathology on miRNA (miR-106b) expression (Wang et al., 2010), while miR-106b was also found to regulate APP mRNA levels (Hebert et al., 2009). Integrating all miRNA data it seems that the miRNA network in AD is tightly regulated by feedback loops (Schonrock et al., 2011). An ultimate challenge will be an integrated view of APP/Aβ regulation that takes into account the role of miRNAs, mRNA transcription, translation, post-translational modifications, subcellular compartmentalization, brain regional differences, and changes over time, both under physiological and pathological conditions. This will determine whether miRNAs are merely fine-tuning instruments as far as the major players in AD are concerned or whether they indeed play a critical role in pathogenesis and disease progression.

## Outlook—where is the field heading and will functional genomics contribute to finding a cure?

What does the future hold in store? As post-translational modifications are crucial for the brain's functioning (and in line with “kinases” being a focus of this special “Frontiers” issue) it will be important to move from the general proteome to the phospho-proteome, glyco-proteome and so forth as these modifications determine where the proteins are subcellularly targeted, with which proteins they interact and what their activities are. It will also be important (and as exemplified by transcriptomics) to be able to work with increasingly smaller amounts of starting material to determine how individual cells respond differentially to toxic insults and how this leads to neurodegenerative changes in some cells, and not the others. Although laser capturing allows enriching cells of the same subtype one would ultimately like to work with single cells. Another aspects not discussed here is that the standard strategies do not discriminate between a protein that has been synthesized quite a while ago and one that has been synthesized as a consequence of a recent toxic insult. Here, novel methods of click chemistry that allow to identify *de novo* synthesized proteins using non-canonical amino acids will be helpful (Dieterich et al., 2006, 2010).

Recent advances in sequencing technologies and assembly algorithms have facilitated the reconstruction of the entire transcriptome by deep RNA sequencing, and with costs going down, these methods will certainly be more widely applied but they will also heavily depend on bioinformatics (Martin and Wang, 2011). Neither proteins nor RNAs act in isolation and therefore major efforts will go in determining the cellular interactome under normal and specific neurodegenerative conditions, including the role of miRNAs and the interaction between RNAs in general as well as proteins (Konig et al., 2011). A major challenge will be in integrating the proteomics and transcriptomics findings obtained in various cellular and animal systems as well as the human brain. It is remarkable however that despite differences in species and significant postmortem delays in humans, one finds a remarkable overlap of deregulated genes and proteins when comparing datasets obtained in human AD brains with those of brains of AD mouse models. This is comforting, as it would indicate that the functional genomics approaches in the more accessible experimental cellular and animal systems generate data that are meaningful for the human condition. It is also remarkable to find that functional genomics confirms the known suspects, both at the level of the specific gene or protein, and at the level of the deregulated category or signaling pathway. On top of that however, functional genomics has been instrumental in identifying new players, genes and proteins that mediate or ameliorate neurotoxicity and of miRNAs as fine-regulating tools of gene transcription. Besides from understanding pathogenic mechanisms, functional genomics has its merits furthermore in identifying biomarkers that can be employed as diagnostic tools for neurodegenerative disorders such as AD. Together, functional genomics has had a major role in the past in understanding the toxic roles of tau and Aβ in AD and it is expected to have a more important role as the technology becomes more widely applied.

### Conflict of interest statement

The authors declare that the research was conducted in the absence of any commercial or financial relationships that could be construed as a potential conflict of interest.

## Research Topic:

From structural to molecular systems biology: experimental and computational approaches to unravel mechanisms of kinase activity regulation in cancer and neurodegeneration

## Topic Editor:

Matteo Barberis, Humboldt University Berlin, Germany; Max Planck Institute for Molecular Genetics, Berlin, Germany
